# Increased plasma proline concentrations are associated with sarcopenia in the elderly

**DOI:** 10.1371/journal.pone.0185206

**Published:** 2017-09-21

**Authors:** Kenji Toyoshima, Marie Nakamura, Yusuke Adachi, Akira Imaizumi, Tomomi Hakamada, Yasuko Abe, Eiji Kaneko, Soiciro Takahashi, Kentaro Shimokado

**Affiliations:** 1 Department of Geriatric and Vascular Medicine, Tokyo Medical and Dental University Graduate School, Tokyo, Japan; 2 Institute for Innovation, Ajinomoto, Corartion, Incprporated, Kawasaki-shi, Kanagawa, Japan; 3 Mituke Municipal Hospital, Mistuke-shi, Niigata, Japan; Ehime University Graduate School of Medicine, JAPAN

## Abstract

**Background and purpose:**

Metabolome analyses have shown that plasma amino acid profiles reflect various pathological conditions, such as cancer and diabetes mellitus. It remains unclear, however, whether plasma amino acid profiles change in patients with sarcopenia. This study therefore aimed to investigate whether sarcopenia-specific changes occur in plasma amino acid profiles.

**Methods:**

A total of 153 community-dwelling and seven institutionalized elderly individuals (56 men, 104 women; mean age, 77.7±7.0 years) were recruited for this cross-sectional analysis. We performed a comprehensive geriatric assessment, which included an evaluation of hand grip strength, gait speed, muscle mass and blood chemistry, including the concentration of 18 amino acids.

**Results:**

Twenty-eight of the 160 participants met the criteria for sarcopenia established by the Asian Working Group on Sarcopenia in Older People. Univariate analysis revealed associations between the presence of sarcopenia and a higher plasma concentration of proline and glutamine, lower concentrations of histidine and tryptophan. Multivariable analysis revealed that a higher concentration of proline was the only variable independently associated with sarcopenia.

**Conclusions:**

The plasma concentration of proline may be useful for understanding the underlying pathophysiology of sarcopenia.

## Introduction

Sarcopenia is the age-associated loss of skeletal muscle mass, strength, and physical performance [1,2]. It affects the mortality, cognitive function, and quality of life of elderly people [3]. The prevalence of sarcopenia is reported to be 11.8–52.9%, depending on variables such as age, sex, and social and nutritional status [4–6]. The prevalence of sarcopenia is expected to rise with the rapid increase of life-span worldwide.

However, the molecular mechanisms underlying sarcopenia remain largely unknown, and diagnosis requires time-consuming measurements. Therefore, it is important to elucidate the pathophysiology and to develop modalities for the early and precise diagnosis of this condition.

Amino acid profiling and metabolome analysis using mass spectrometry have been powerful analytical tools [7] for revealed associations between plasma amino acid profiles and various diseases, including cancer [8–10], obesity and glucose metabolism [11–14], end-stage liver disease [15], inflammatory bowel disease [16], depression [17], rheumatoid arthritis [18], acute dissecting aortic aneurysm [19], and aging and longevity [20, 21]. Understanding changes in amino acid profiles may therefore be useful for early diagnosis and insight into the pathophysiological mechanisms underlying such diseases.

Recently, changes in plasma amino acid profiles have been reported to be associated with muscle mass in functionally limited elderly people [22], poor muscle quality in elderly people [23], and sedentary lifestyles [24]. It is not clear, however, whether there is any amino acid profiles specificity for sarcopenia.

We therefore investigated the plasma amino acid profiles of elderly people living in a rural area of Japan.

## Materials and methods

### Ethics

This study was conducted in accordance with the Declaration of Helsinki. The protocol was approved by the ethics committees of Tokyo Medical and Dental University (#2146) and the Ajinomoto Corporation Institution. Written informed consent obtained from all participants or their family members if their cognitive function was impaired.

### Participants and study design

In this cross-sectional study, 153 community-dwelling and seven institutionalized elderly people (56 men, 104 women; mean age, 77.7±7.0 years), were recruited from a rural town in Japan (Mitsuke city, Niigata Prefecture) between January 2015 and June 2016. The participants were asked to answer questionnaires regarding their activities of daily living (ADL), instrumental activities of daily living (IADL), medical history and medication in advance of their physical checkups. In the morning, fasting blood was collected and physical checkups and tests for sarcopenia were conducted.

### Sarcopenia

Sarcopenia was diagnosed in accordance with the Asian Working Group for Sarcopenia consensus panel definitions [25] by measuring hand grip strength, gait speed, and skeletal muscle index (SMI). Hand grip strength was measured in both hands using a digital hand dynamometer (TKK 5401 Grip-D; Takei Scientific Instruments Company Limited, Niigata, Japan). Gait speed was assessed using a 6-m walking test. The participants were asked to walk 10 m at a normal speed, and the walking time for 6 m in between was measured using a stopwatch. Whole body skeletal muscle mass was measured by segmental multi-frequency bioelectrical impedance analysis using a body composition analyzer (Inbody 770; Biospace Co., Ltd., Seoul, Korea). SMI was calculated by dividing appendicular skeletal muscle mass by height squared in meters (kg/m^2^).

### Biochemical analysis

Blood samples were collected after overnight fasting. Plasma was prepared within 6 h and stored at –80°C until analysis. The concentrations of 18 human proteinogenic amino acids in plasma were measured using high-performance liquid chromatography-electrospray ionization-tandem mass spectrometry, as previously reported [26]. Plasma concentrations of aspartate and cysteine were excluded from analysis because they are not stable in blood under our analytical conditions.

Measurement of following items was conducted at a commercial laboratory (SRL Co., Ltd., Tokyo, Japan) using overnight fasting serum samples: total protein (TP), albumin (Alb), aspartate aminotransferase (AST), alanine aminotransferase (ALT), gamma-glutamyltransferase (γGTP), blood urea nitrogen (BUN), creatinine (Cre), immune reactive insulin (IRI), HbAlc, and plasma glucose. For the assessment of insulin resistance, homeostasis model assessment of insulin resistance (HOMA-IR) was calculated as follows: fasting plasma glucose (mg/dl) × IRI (IU/ml)/405

### Comprehensive geriatric assessment

The Barthel index was used to assess ADL [27], the IADL Scale to assess IADL [28], the Mini-Mental State Examination (MMSE) to assess cognitive function [29], and the Geriatric Depression Scale-15 (GDS-15) to assess depression [30].

### Statistical analysis

Continuous variables with normal distribution such as age and body mass index (BMI) were presented as mean ± standard deviation. Welch’s t-test was used to compare these variables between the sarcopenia and no sarcopenia groups. Continuous variables with non-normal distributions, such as amino acid and albumin concentrations, were presented as median and range. The Mann–Whitney U test was used for group comparisons, and the chi-square test was used for categorical variables. P<0.05 was considered to indicate statistical significance. Multivariable logistic regression analysis was used to identify independent factors associated with sarcopenia, including all variables that were found to be statistically significant in the univariate analyses. Odds ratios (ORs) with 95% confidence intervals (95% CIs) were also calculated. All statistical analyses were performed using Stata version 14 (Stata Corp., College Station, TX, USA).

## Results

### Characteristics of the study participants

Of the 160 total participants, 28 (17.5%) were sarcopenic. Among the 153 community-dwelling participants, 21 (13.7%) were sarcopenic, while all seven participants living at a nursing home were sarcopenic. All nursing home residents were unable to walk 6 m, had poor grip strength, and had clinically apparent reductions in muscle mass. Compared with the non-sarcopenic participants, the sarcopenic participants were older (p<0.001), had lower BMI (p = 0.005), more frequently suffered from dementia (p<0.0001), more frequently had impaired ADL (p<0.0001) and IADL (p<0.001), and were more depressive (p = 0.0001) (Table 1). No differences in were observed between the sarcopenia and no sarcopenia groups in sex or comorbidities, including hypertension, diabetes, and dyslipidemia. Regarding blood chemistry, sarcopenic participants had lower concentrations of TP, Alb, and ALT. Plasma glucose concentrations of the sarcopenic participants were slightly but significantly lower than those of the non-sarcopenic participants; however, no differences were seen in HbA1c or HOMA-IR.

**Table 1:** Characteristics of the study participants.

Characteristics		All (n = 160)	Sarcopenia (n = 28)	No sarcopenia (n = 132)	p-value
Age, years^a^^,^^d^		77.7±7.0	85.1±4.8	76.1±6.3	<0.0001***
Sex^b^^,^^f^					0.93
	Female	104	18	86	
	Male	56	10	46	
Community-dwelling vs. nursing home residents ^f^		153 vs. 7	21 vs. 7	131 vs. 0	<0.0001***
BMI ^a^^,^^d^		22.5±3.0	20.7±2.9	22.8±2.9	0.0054**
Barthel index		100 (0–100)	100 (0–100)	100 (95–100)	<0.0001***
IADL		1 (0–1)	0.8 (0–1)	1 (0.375–1)	<0.0001***
MMSE^c^^,^^e^		28 (0–30)	24.5 (0–30)	28 (21–30)	<0.0001***
GDS 15^c^^,^^e^		2 (0–14)	4 (1–13)	1.5 (0–14)	0.0001***
Hypertension^b^^,^^f^		104	17	87	0.6
Diabetes^b^^,^^f^		37	5	32	0.47
Dyslipidemia^b^^,^^f^		78	9	69	0.053
Laboratory data					
	TP (g/dl)^c^^,^^e^	7.3 (5.6–8.6)	7.2 (5.6–8.2)	7.4 (6.6–8.6)	0.0318*
	Alb (g/dl)^c^^,^^e^	4.4 (2.4–5.3)	4.0 (2.4–4.8)	4.4 (3.5–5.3)	<0.0001***
	AST (IU/l)^c^^,^^e^	23 (12–56)	21 (15–56)	24 (12–44)	0.1288
	ALT (IU/l)^c^^,^^e^	16 (6–70)	13 (6–70)	17 (9–52)	0.0002***
	γGTP (IU/l)^c^^,^^e^	21 (10–108)	19 (10–42)	22 (10–108)	0.1096
	BUN (mg/dl)^c^^,^^e^	15.9 (6.9–38.2)	15.4 (6.9–38.2)	16 (7.1–34.3)	0.8158
	Cre (mg/dl)^c^^,^^e^	0.69 (0.43–2.54)	0.7 (0.43–1.59)	0.69 (0.44–2.54)	0.656
	Glucose (mg/dl)^c^^,^^e^	99 (72–218)	93.5 (72–218)	100 (80–170)	0.0366*
	IRI (μU/ml)^c^^,^^e^	4.5 (0.58–36.4)	3.7 (4.9–8)	4.63 (0.58–19.1)	0.1637
	HOMA-IR^c^^,^^e^	1.09 (0–19.59)	0.825 (0.16–19.59)	1.14 (0–6.55)	0.1079
	HbA1c (%)^c^^,^^e^	5.9 (4.8–8.0)	5.7 (4.9–8.0)	5.9 (4.8–7.7)	0.1112

^a^ Expressed as mean±standard deviation.

^b^ Expressed as number.

^c^ Expressed as median (range).

^d^ p-values were calculated using Welch’s t-test.

^e^ p-values were calculated using the Mann–Whitney U test.

^f^ p-values were calculated using the chi-square test.

* p<0.05,

**p<0.01,

***p<0.001.

Hypertension: systolic blood pressure ≥140 mmHg, diastolic blood pressure ≥90 mmHg, or on medication; Diabetes: HbA1c ≥6.5%, fasting plasma glucose ≥126 mg/dl, or on medication; Dyslipidemia: on medication (cholesterol was not measured in this study); BMI, body mass index; IADL, instrumental activities of daily living; MMSE, Mini-Mental State Examination; TP, total protein; Alb, albumin, AST, aspartate transaminase; ALT, alanine transaminase; γGTP, γ-glutamyltransferase; BUN, blood urea nitrogen; Cre, creatinine; IRI, immune reactive insulin; HOMA-IR, homeostasis model assessment of insulin resistance.

### Amino acid profiles: Univariate and multivariate analysis

Univariate analysis showed that the sarcopenic participants had lower concentrations of histidine (p = 0.0025) and tryptophan (p = 0.0012) and higher concentrations of glutamine (p = 0.0177) and proline (p = 0.0096) than the non-sarcopenic participants (Table 2).

**Table 2:** Plasma amino acid profiles of the sarcopenic and non-sarcopenic groups.

Amino acid	Sarcopenia (n = 28)	No sarcopenia (n = 130)	p-value
**Ala**	350.2 (219.9–639.6)	345.4 (199.1–658.5)	0.5295
**Arg**	99.7 (58.5–145.3)	94.0 (46.7–148.6)	0.5034
**Asn**	46.1 (29.2–75.0)	45.7 (33.9–76.0)	0.8188
**Asp**	Not tested	Not tested	Not tested
**Cys**	Not tested	Not tested	Not tested
**Gln**	629.8 (507.8–944.9)	596.3 (445.8–780.9)	0.0177*
**Glu**	24.3 (9.2–60.9)	28.1 (10.5–76.7)	0.2693
**Gly**	209.7 (170.7–377.8)	221.9 (116.1–522.7)	0.9071
**His**	75.1 (49.7–106.1)	80.8 (57.7–129.5)	0.0025**
**Ile**	51.9 (36.2–113.2)	55.2 (35.7–110.3)	0.9356
**Leu**	97.6 (60.1–201.1)	108.4 (72.8–211.3)	0.1384
**Lys**	184.2 (119.2–304.7)	190.8 (117.4–323.4)	0.1129
**Met**	24.3 (15.8–34.2)	25.0 (16.0–40.2)	0.3549
**Phe**	59.2 (45.8–84.1)	60.1 (44.4–94.9)	0.9356
**Pro**	163.6 (75.4–540.6)	130.3 (80.8–335.4)	0.0096**
**Ser**	100.3 (62.9–149.5)	112.2 (60.2–196.6)	0.069
**Thr**	108.9 (44.7–153.0)	118.9 (66.3–215.5)	0.0502
**Trp**	42.0 (23.4–62.7)	50.1 (27.9–85.5)	0.0012**
**Tyr**	62.7 (44.0–86.1)	63.5 (42.1–99.7)	0.5503
**Val**	192.6 (113.0–327.6)	205.5 (128.9–349.0)	0.1268

Expressed as median (range).

p-values were calculated using the Mann–Whitney U test.

* p<0.05,

**p<0.01

AA, amino acids; Glu, glutamic acid; Gly, glycine; His, histidine; Ile, isoleucine; Leu, leucine; Lys, lysine; Met, methionine; Phe, phenylalanine; Pro, proline; Ser, serine; Thr, threonine; Trp, tryptophan; Tyr, tyrosine; Val, valine.

Asp and Cys were not tested in this analysis because they are unstable in blood.

Multiple logistic regression analysis including all factors showing significant differences in Tables 1 and 2 as explanatory factors revealed that the following parameters were significantly associated with sarcopenia: age (OR: 1.21; 95% CI: 1.05–1.39); MMSE scores (OR: 0.69; 95% CI: 0.48–0.98); BMI (OR: 0.71; 95% CI:0.51–1.00); and Barthel index (OR: 0.35; 95% CI: 0.16–0.77). Regarding amino acids, only plasma proline remained an independent risk factor for sarcopenia (OR: 1.02; 95% CI: 1.00–1.03) (Table 3). The higher plasma concentration of proline was significant even when the analysis was conducted excluding the seven institutionalized participants, who all had sarcopenia.

**Table 3:** Multiple logistic regression analysis of factors associated with sarcopenia.

Variable	OR	95% CI	p-value
**Age**	1.21	1.05–1.39	0.009*
**MMSE**	0.69	0.48–0.980	0.039*
**BMI**	0.71	0.51–1.00	0.049*
**Barthel index**	0.35	0.16–0.77	0.009**
**IADL**	18.43	0.00–192476	0.537
**GDS**	1.28	0.95–1.73	0.104
**Alb**	0.26	0.0.-2.25	0.221
**ALT**	0.91	0.75–1.10	0.327
**glucose**	1.01	0.97–1.05	0.655
**Gln**	1.00	0.98–1.01	0.605
**His**	0.98	0.89–1.07	0.629
**Pro**	1.02	1.00–1.03	0.028*
**Trp**	1.02	0.93–1.12	0.732

OR, odds ratio; CI, confidence interval.

MMSE, Mini-Mental State Examination; BMI, body mass index; IADL, instrumental activities of daily living; Alb, albumin; ALT, alanine transaminase; Gln, glutamine; His, histidine; Pro, proline; Trp, tryptophan.

* p<0.05,

**p<0.01

## Discussion

Based on the results of the present study, a higher plasma concentration of proline was found to be associated with sarcopenia in the elderly. This association remained significant even when the analysis was conducted with only the community-dwelling participants. To our knowledge, this is the first study to report an association between plasma amino acid profiles and sarcopenia.

Our findings support those reported in previous studies. Fukai et al. reported that lower physical activity and longer sitting hours were associated with higher plasma concentrations of proline, as well as branched-chain amino acids and alanine [24]. In our study, plasma alanine concentrations were higher in sarcopenic than in non-sarcopenic elderly people; however, this difference was not statistically significant. Although the study by Fukai et al. included younger people and sarcopenia was not evaluated, their findings are in agreement with ours, probably because it is well-known that a sedentary lifestyle accelerates the development of sarcopenia. Ribel-Madsen et al. found that young, healthy low-birth-weight men showed high plasma concentrations of proline and alanine after high-fat overfeeding, probably due to an increase in insulin-resistance and proteolysis in skeletal muscle [31]. Ilaiwy et al. observed that muscle cell atrophy was associated with increased concentrations of proline and alanine together with other metabolites including glutamine in culture media *in vitro* [32], again agreeing with our findings.

Toshima et al. previously reported that before liver transplantation, the plasma concentration of glutamine was significantly lower in patients with than in patients without sarcopenia [33]. The result is inconsistent with our study. We defined sarcopenia as a loss of muscle mass and dysfunction of the remaining muscle. However, Toshima et al. defined sarcopenia as a loss of muscle mass in the area of the major psoas muscle; furthermore, they did not assess muscle functions such as grip power or gait speed. In addition, their study population was younger than ours (55 vs. 77 years). Glutamine metabolism changes with age [34], and liver dysfunction was the most prevalent disorder among their study population. The liver has a key role in glutamine metabolism [35]. These factors are may explain why their results were than ours.

In the present study, no significant changes were observed in the concentrations of branched-chain amino acids. By contrast, many previous studies reported finding changes in plasma levels of branched-chain amino acids such as leucine and valine in relation to sarcopenia. For example, Lustgarten et al. reported that branched-chain amino acids and their metabolites were associated with thigh muscle cross-sectional area [22]. On the other hand, Moaddel et al. found that a low muscle strength to mass ratio was associated with higher concentrations of leucine, isoleucine, tryptophan, serotonin, and methionine [23]. In our study, tryptophan was significantly lower in sarcopenic participants, but this was not an independent risk for sarcopenia according to multivariate analysis, probably due to its close association with age. One factor that may have affected the discrepancies between those reports was differences in the study design and participants. Some studies recruited participants aged 65 years or older, while others included participants with a wider age range. In addition, we compared sarcopenic and non-sarcopenic individuals, whereas other studies investigated the relationship between components of sarcopenia and metabolic changes. The components of sarcopenia—muscle mass, grip strength, and gait speed—do not change in parallel with its progression. In fact, among the community-dwelling sarcopenic elderly in our study, only 19% had decreased gait speed; in addition, 76% of the non-sarcopenic elderly with decreased muscle mass met the criteria for sarcopenia. Other factors that might affect this discrepancy include differences in race, environment, lifestyle, and eating habits [24, 35–37].

The pathophysiological significance of our findings for the mechanisms underlying the increase in plasma proline concentrations remains to be elucidated. One possibility is that higher plasma concentrations lead to sarcopenia. A high concentration of proline has been shown to induce oxidative damage to protein, lipids, and DNA in rats [38]. Proline prevents the uptake of glutamate, a neurotransmitter, by neurons in the cerebral cortex and hippocampus of rats [39]. Plasma proline concentrations are increased in patients with Alzheimer’s disease and amnestic mild cognitive impairment [40]. The findings of these previous studies suggest the possibility that increased proline concentrations induce sarcopenia either directly or indirectly through cognitive function decline.

Another more likely possibility is that a high proline level reflects metabolic changes secondary to sarcopenia. Proline, alanine and glutamine are used as a source of energy metabolism by feeding the anaplerotic pathway of the tricarboxylic acid cycle. In malnourished patients, proline and alanine are generated by proteolysis in skeletal muscle and used for energy metabolism. By contrast, in healthy elderly individuals with sufficient carbohydrate intake, proline and alanine are not metabolized via the anaplerotic pathway, resulting in the higher concentrations of those amino acids. However, these proposed mechanisms are still speculative and need to be delineated.

This study did have some limitations. First, selection bias cannot be ruled out. The participants were volunteers and not randomly selected, so this might have affected some findings, as there were more women than men, and there were fewer participants than expected with dementia according to the overall incidence of dementia in the elderly population in Japan. Racial and regional factors might also have influenced the results because this study was conducted in a single Japanese rural city. Second, while the incidence of sarcopenia was similar to those reported for the general populations in Japan and other countries, the number of sarcopenic elderly in the present study was relatively small. This might have influenced the power to detect some changes in plasma amino acid levels. Finally, because this was a cross-sectional study, we could not correlate our findings with the prognosis of sarcopenia in the elderly.

In conclusion, we found that a high plasma concentration of proline is an independent risk factor for sarcopenia. The results of the present study, along with those from other recent reports, suggest that further research on the pathophysiological and clinical significance of plasma amino acids profile among the sarcopenic elderly is warranted.
